# Mind-body therapies for pro-inflammatory cytokines in patients with depression: findings from a systematic review of randomized controlled trials

**DOI:** 10.3389/fimmu.2025.1677872

**Published:** 2025-10-21

**Authors:** Zhengyang Mei, Shi Luo, Chenyi Cai, Chifong Lam, Tingfeng Wang, Haichang Jia, Longjiang Chen, Ranran He

**Affiliations:** ^1^ School of Physical Education, Southwest University, Chongqing, China; ^2^ Key Laboratory of Cognition and Personality, Faculty of Psychology, Ministry of Education, Southwest University, Chongqing, China; ^3^ School of Physical Education, Shanghai University of Sport, Shanghai, China; ^4^ School of Physical Education, Yunnan Normal University, Yunnan, China; ^5^ Department of Physical Education, Tianjin University, Tianjin, China

**Keywords:** mind-body therapies, inflammatory markers, inflammation, depression, systematic review

## Abstract

**Objective:**

Depression is one of the most common mental disorders and is the leading cause of disability worldwide. The objective of this systematic review was to synthesize the latest evidence from randomized controlled trials (RCTs) regarding the effectiveness of mind-body therapies (MBTs) on pro-inflammatory cytokines in patients with depression.

**Methods:**

A literature search was conducted in five electronic databases—PubMed, Embase, Web of Science, EBSCOhost, and Scopus. The quality of the included studies was evaluated using the Revised Cochrane risk-of-bias tool for randomized trials (RoB 2). A narrative synthesis of the included studies was conducted.

**Results:**

The 12 RCTs provided 21 pieces of evidence involving a total of 1,058 patients with depression. The risk of bias among the included studies ranged from low to high, with 4 studies assessed as low risk, 4 as some concerns, and 4 as high risk. Among the 21 pieces of evidence evaluated, 14 supported the positive impact of MBTs on pro-inflammatory cytokine levels in patients with depression.

**Conclusion:**

MBTs have been widely recognized in nursing for their low risk and substantial benefits, and they hold promise as a complementary therapy to improve physiological health outcomes in patients with depression. However, the studies included commonly exhibit potential limitations in terms of intervention materials, adherence, and outcome measures. It is suggested that future research should further examine the existing evidence to strengthen the empirical foundation for incorporating MBTs into nursing care for depression.

**Systematic review registration:**

https://www.crd.york.ac.uk/prospero/, identifier CRD420251113095.

## 1 Background

Depression is one of the most common mental disorders and is the leading cause of disability worldwide (1–3). The clinical characteristics of depression primarily include a depressed mood, diminished interest or pleasure in activities, reduced ability to think or concentrate, and feelings of worthlessness or guilt (4). In recent years, the global prevalence of depression has been steadily increasing, placing a substantial disease burden on society (5, 6). Additionally, depression has been identified as an important risk factor for various physical illnesses, including coronary artery disease (7), sleep disorders (8), and cognitive impairments (9).

Pharmacological treatment is a common approach for alleviating depression; however, its therapeutic effects typically take at least four weeks to manifest, and during this period, patients may experience side effects such as headaches, anxiety, and agitation (10). Several complementary therapies have been developed to effectively address this challenge. Among them, mind-body therapies (MBTs) have become an important approach for alleviating depression due to their lower treatment risks and higher potential for efficacy (11). MBTs are rooted in ancient Eastern traditions and aim to enhance overall well-being by harnessing the interplay between the mind, body, and spirit (12, 13). The efficacy of MBTs in alleviating depression in both clinical and non-clinical populations has been supported by numerous studies (14–19).

Recent research suggests that the potential mechanism through which MBTs exert their antidepressant effects is related to the body’s inflammatory response (20, 21), primarily mediated by cytokines such as interleukin-1β (IL-1β), interleukin-6 (IL-6), and tumor necrosis factor-α (TNF-α) (22–24). Compared to healthy individuals, patients with depression may exhibit higher levels of pro-inflammatory cytokines. MBTs can reduce the levels of inflammatory markers by modulating the hypothalamic-pituitary-adrenal (HPA) axis and the autonomic nervous system (ANS) (20). For example, meditation can alter the brain’s neural response to stress or threats, and these changes may be associated with a reduction in the levels of inflammatory markers such as IL-6 (25). Yoga has been found to effectively reduce stress and improve cognition, benefits that are particularly important for reducing pro-inflammatory responses (26, 27). However, the above evidence primarily focuses on other clinical populations, and it remains uncertain whether it is applicable to patients with depression.

To the best of our knowledge, no study has yet comprehensively evaluated the overall effectiveness of MBTs in improving pro-inflammatory cytokine levels in patients with depression. Applying a complementary therapy that offers both low risk and substantial benefits to improve physiological health outcomes in patients with depression lays the foundation for subsequent treatment and promotes the comprehensive recovery of physical and mental health. Given the aforementioned evidence gap, this systematic review aimed to synthesize the latest evidence from randomized controlled trials (RCTs) regarding the effectiveness of MBTs on pro-inflammatory cytokines in patients with depression.

## 2 Methods

This systematic review adhered to the Preferred Reporting Items for Systematic Reviews and Meta-Analyses (PRISMA) 2020 (28) and was registered in the International Prospective Register of Systematic Reviews (PROSPERO) under the registration number CRD420251113095.

### 2.1 Search methods

We conducted a literature search in five electronic databases—PubMed, Embase, Web of Science, EBSCOhost, and Scopus—using the Boolean algorithm established for this systematic review. In addition, we manually searched Google Scholar and reference lists of studies with similar designs to ensure the comprehensiveness of the literature search. The literature search covered the period from the inception of each database to June 2025. The search strategy is presented in **Table 1**, per the PubMed database.

**Table 1:** PubMed search strategy.

Order	Boolean operator
1	(Depression disorder or Depression[MeSH Terms]) OR (Depression disorder[Title/Abstract] OR Depression[Title/Abstract] OR Depress*[Title/Abstract] OR Melancho*[Title/Abstract] OR Mood*[Title/Abstract] OR Emotion*[Title/Abstract])
2	Mind body*[Title/Abstract] OR Mind-body*[Title/Abstract] OR Mindfulness[Title/Abstract] OR Meditation[Title/Abstract] OR Martial Arts[Title/Abstract] OR Arts, Martial[Title/Abstract] OR Kung Fu[Title/Abstract] OR Gongfu[Title/Abstract] OR Gong Fu[Title/Abstract] OR Fu, Gong[Title/Abstract] OR Wushu[Title/Abstract] OR Shadow boxing[Title/Abstract] OR Tai Ji[Title/Abstract] OR Tai-ji[Title/Abstract] OR Tai Chi[Title/Abstract] OR Chi, Tai[Title/Abstract] OR Tai Ji Quan[Title/Abstract] OR Ji Quan, Tai[Title/Abstract] OR Quan, Tai Ji[Title/Abstract] OR Taiji[Title/Abstract] OR Taijiquan[Title/Abstract] OR T'ai Chi[Title/Abstract] OR Tai Chi Chuan[Title/Abstract] OR Qigong[Title/Abstract] OR Qi Gong[Title/Abstract] OR Ch'i Kung[Title/Abstract] OR Baduanjin[Title/Abstract] OR Yoga[Title/Abstract] OR Pilates[Title/Abstract] OR Exercise Movement Techniques[Title/Abstract] OR Movement Techniques, Exercise[Title/Abstract] OR Exercise Movement Technics[Title/Abstract] OR Pilates-Based Exercises[Title/Abstract] OR Exercises, Pilates-Based[Title/Abstract] OR Pilates Based Exercises[Title/Abstract] OR Pilates Training[Title/Abstract] OR Training, Pilates[Title/Abstract] OR Tae Kwon Do[Title/Abstract] OR Judo[Title/Abstract] OR Karate[Title/Abstract] OR Aikido[Title/Abstract] OR Jujitsu[Title/Abstract]
3	Interleukin-1[Title/Abstract] OR Interleukin-1β[Title/Abstract] OR Interleukin-1beta[Title/Abstract] OR IL-1[Title/Abstract] OR IL-1β[Title/Abstract] OR IL-1beta[Title/Abstract] OR Interleukin 1[Title/Abstract] OR Interleukin 1β[Title/Abstract] OR Interleukin 1beta[Title/Abstract] OR IL 1[Title/Abstract] OR IL 1β[Title/Abstract] OR IL 1beta[Title/Abstract] OR Interleukin-6[Title/Abstract] OR IL-6[Title/Abstract] OR Interleukin 6[Title/Abstract] OR IL 6[Title/Abstract] OR Tumor necrosis factor[Title/Abstract] OR Tumor necrosis factor-α[Title/Abstract] OR Tumor necrosis factor-alpha[Title/Abstract] OR TNF[Title/Abstract] OR TNF-α[Title/Abstract] OR TNF-alpha[Title/Abstract] OR Tumor necrosis factor α[Title/Abstract] OR Tumor necrosis factor alpha[Title/Abstract] OR TNF α[Title/Abstract] OR TNF alpha[Title/Abstract]
4	(Randomized controlled trial[Publication Type]) OR (Randomized[Title/Abstract] OR Placebo[Title/Abstract])
5	1 AND 2 AND 3 AND 4

### 2.2 Inclusion and exclusion criteria

The inclusion and exclusion criteria for this systematic review strictly followed the Population, Intervention, Comparator, Outcome, and Study design (PICOS) framework. Regarding the inclusion criteria, the population was restricted to patients with depression (age ≥ 18 years); the intervention was limited to MBTs; the comparator included both non-active controls (e.g., no exercise, wait-list) and active controls (e.g., treatment as usual, standard care, placebo); the outcome focused on pro-inflammatory cytokines, including IL-1β, IL-6, and TNF-α; and the study design was restricted to RCTs. Studies targeting non-depressed patients, non-MBTs, and non-RCTs were excluded. Data from the same group of subjects were only included in a single study that provided more comprehensive information.

### 2.3 Study selection and quality appraisal

According to the inclusion and exclusion criteria, two independent researchers conducted literature screening using EndNote 20.6 reference management software. After removing duplicates, the remaining records were screened sequentially based on their titles, abstracts, and full texts, with the reasons for exclusion systematically documented for each record. Two independent researchers evaluated the quality of the included studies using the Revised Cochrane risk-of-bias tool for randomized trials (RoB 2) (29). Any disagreements arising during this process were resolved through consultation with a third author.

### 2.4 Data extraction and synthesis

Upon identifying studies that met the inclusion criteria, two independent researchers extracted the following information from each included study: sociodemographic characteristics, intervention and comparator, implementation parameters, and outcome. A narrative synthesis of the included studies was conducted. We utilized the Template for Intervention Description and Replication (TIDieR) checklist to evaluate the adequacy of intervention reporting (30).

## 3 Results

### 3.1 Search outcomes

**Figure 1:**
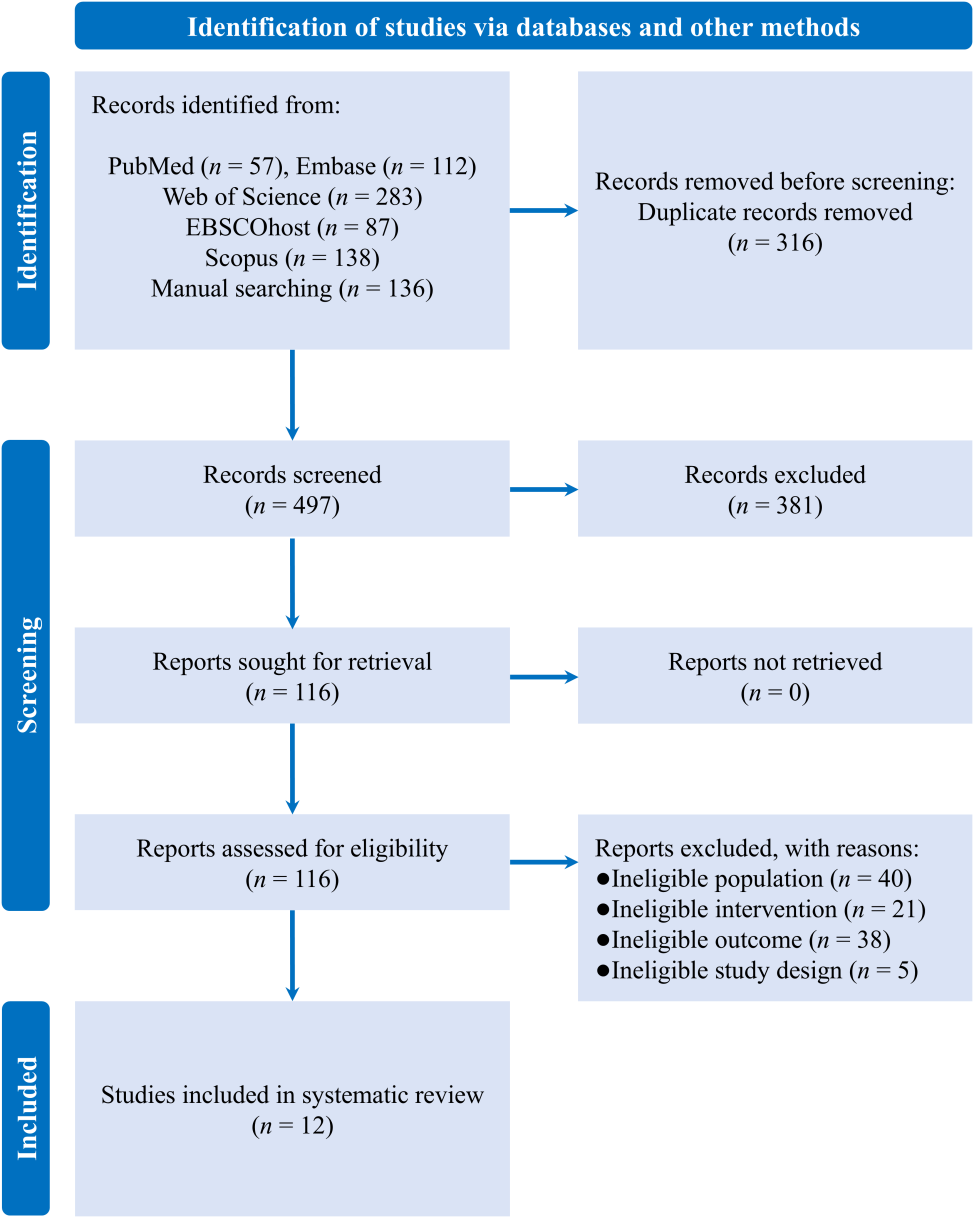
PRISMA flow diagram.

A literature search of various databases yielded a total of 813 records, of which 316 were duplicates. After screening the remaining records, 12 eligible RCTs were included in the systematic review (see **Figure 1**) (31–42).

### 3.2 Study characteristics and quality appraisal

**Figure 2:**
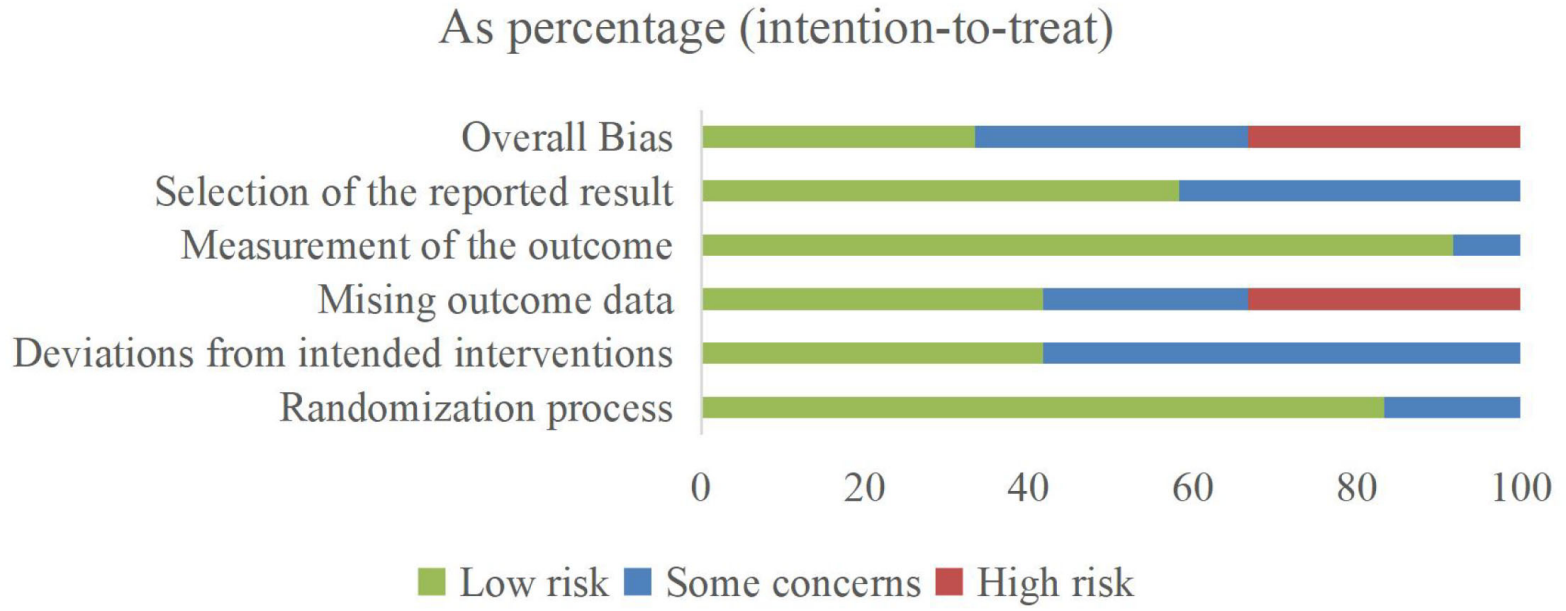
Risk of bias summary.

Evidence regarding the effectiveness of MBTs on pro-inflammatory cytokine levels in patients with depression was distributed across multiple countries, including Australia (one trial), Brazil (one trial), China (four trials), India (one trial), Sweden (one trial), Thailand (one trial), and the United States (three trials). The evidence involved 1,058 patients with depression, ranging in age from 18 to 82 years. The interventions included Baduanjin, mindfulness, meditation, Tai Chi, yoga, and combined forms of MBTs. Regarding implementation parameters, the included studies had an average intervention period of 8.6 weeks, an average frequency of 3.3 sessions per week, and an average duration of 82.5 minutes. The controls included active controls, no exercise, treatment as usual, and wait-list. In terms of outcome measures, evidence for IL-1β was reported in 4 studies, IL-6 in 11 studies, and TNF-α in 6 studies. The main characteristics of the included RCTs are presented in **Table 2**. The risk of bias among the included studies ranged from low to high, with 4 studies assessed as low risk, 4 as some concerns, and 4 as high risk (see **Figure 2**; **Table 3**).

**Table 2:** Main characteristics of included randomized controlled trials.

Study ID	Country	Age (Mean ± SD)	Sample size (Male)	Intervention	Comparator	Outcome
Ewais et al. (2021) (31)	Australia	T: 22.0 ± 3.0 C: 22.0 ± 4.0	T: 33 (14) C: 31 (10)	Mindfulness. Period: 8; Frequency: 1; Duration: 120	Treatment as usual	IL-6
Liu et al. (2024a) (32)	China	19 to 29	T: 26 (7) C: 30 (10)	Mindfulness. Period: 8; Frequency: 1; Duration: 150	Wait-list	IL-1β, IL-6, TNF-α
Liu et al. (2024b) (33)	China	T: 58.9 ± 10.8 C: 56.2 ± 11.5	T: 50 (31) C: 50 (29)	Baduanjin exercise combined with rational emotive behavior therapy. Period: 8; Frequency: 14; Duration: 30	Active control	IL-6
Memon et al. (2017) (34)	Sweden	T: 42.0 ± 11.0 C: 41.0 ± 11.0	T: 81 (14) C: 85 (7)	Mindfulness. Period: 8; Frequency: NR; Duration: NR	Active control	IL-6
Ng et al. (2022) (35)	China	T: 56.0 ± 10.8 C: 54.6 ± 10.2	T: 95 (20) C: 93 (20)	Integrative Body-Mind-Spirit group intervention. Period: 8; Frequency: NR; Duration: NR	Wait-list	IL-1β, IL-6
Nugent et al. (2021) (36)	USA	T: 45.5 ± 12.7 C: 44.8 ± 13.8	T: 48 (4) C: 39 (10)	Yoga. Period: 10; Frequency: 2; Duration: 80	Active control	IL-6, TNF-α
Nyer et al. (2024) (37)	USA	T: 33.1 ± 12.0 C: 33.4 ± 11.8	T: 17 (3) C: 28 (1)	Yoga. Period: 8; Frequency: 2; Duration: 90	Wait-list	IL-1β, IL-6, TNF-α
Prakhinkit et al. (2014) (38)	Thailand	T: 74.0 ± 1.9 C: 81.0 ± 1.7	T: 14 (NR) C: 13 (NR)	Buddhism-based walking meditation. Period: 12; Frequency: 3; Duration: 20-30	No exercise	IL-6
Qiu et al. (2024) (39)	China	T: 38.2 ± 3.6 C: 37.6 ± 3.2	T: 32 (18) C: 32 (17)	Mindfulness. Period: 8; Frequency: 1; Duration: 120	Active control	IL-6, TNF-α
Siddarth et al. (2023) (40)	USA	T: 69.0 ± 6.7 C: 69.5 ± 6.3	T: 85 (26) C: 85 (21)	Tai Chi. Period: 12; Frequency: 1; Duration: 60	Active control	TNF-α
Tolahunase et al. (2018) (41)	India	T: 36.9 ± 8.9 C: 39.1 ± 9.3	T: 29 (13) C: 29 (14)	Yoga- and meditation- based lifestyle intervention. Period: 12; Frequency: 5; Duration: 120	Treatment as usual	IL-6
Torelly et al. (2022) (42)	Brazil	T + C: 37.0 ± 14.3	T + C: 33 (9)	Mindfulness combined with yoga. Period: 1; Frequency: 3; Duration: 30	No exercise	IL-1β, IL-6, TNF-α

Period was measured in weeks, frequency in sessions per week, and duration in minutes.

**Table 3:** Risk of bias summary for the included effect estimates.

Study ID	Outcome	D1	D2	D3	D4	D5	Overall rating
Ewais et al. (2021) (31)	IL-6	Low risk	Low risk	High risk	Low risk	Some concerns	High risk
Liu et al. (2024a) (32)	IL-1β	Low risk	Some concerns	Some concerns	Some concerns	Some concerns	Some concerns
Liu et al. (2024a) (32)	IL-6	Low risk	Some concerns	Some concerns	Some concerns	Some concerns	Some concerns
Liu et al. (2024a) (32)	TNF-α	Low risk	Some concerns	Some concerns	Some concerns	Some concerns	Some concerns
Liu et al. (2024b) (33)	IL-6	Low risk	Low risk	Low risk	Low risk	Low risk	Low risk
Memon et al. (2017) (34)	IL-6	Low risk	Some concerns	High risk	Low risk	Some concerns	High risk
Ng et al. (2022) (35)	IL-1β	Low risk	Low risk	Low risk	Low risk	Low risk	Low risk
Ng et al. (2022) (35)	IL-6	Low risk	Low risk	Low risk	Low risk	Low risk	Low risk
Nugent et al. (2021) (36)	IL-6	Some concerns	Some concerns	Low risk	Low risk	Low risk	Some concerns
Nugent et al. (2021) (36)	TNF-α	Some concerns	Some concerns	Low risk	Low risk	Low risk	Some concerns
Nyer et al. (2024) (37)	IL-1β	Some concerns	Some concerns	High risk	Low risk	Some concerns	High risk
Nyer et al. (2024) (37)	IL-6	Some concerns	Some concerns	High risk	Low risk	Some concerns	High risk
Nyer et al. (2024) (37)	TNF-α	Some concerns	Some concerns	High risk	Low risk	Some concerns	High risk
Prakhinkit et al. (2014) (38)	IL-6	Low risk	Some concerns	Some concerns	Low risk	Low risk	Some concerns
Qiu et al. (2024) (39)	IL-6	Low risk	Low risk	Low risk	Low risk	Low risk	Low risk
Qiu et al. (2024) (39)	TNF-α	Low risk	Low risk	Low risk	Low risk	Low risk	Low risk
Siddarth et al. (2023) (40)	TNF-α	Low risk	Some concerns	High risk	Low risk	Some concerns	High risk
Tolahunase et al. (2018) (41)	IL-6	Low risk	Low risk	Low risk	Low risk	Low risk	Low risk
Torelly et al. (2022) (42)	IL-1β	Low risk	Some concerns	Some concerns	Low risk	Low risk	Some concerns
Torelly et al. (2022) (42)	IL-6	Low risk	Some concerns	Some concerns	Low risk	Low risk	Some concerns
Torelly et al. (2022) (42)	TNF-α	Low risk	Some concerns	Some concerns	Low risk	Low risk	Some concerns

D1, Randomization process; D2, Deviations from intended interventions; D3, Missing outcome data; D4, Measurement of the outcome; D5, Selection of the reported result. Green for "low risk," yellow for "some concerns," and red for "high risk".

### 3.3 Interventions and controls evaluated in the studies

Among the 10 primary domains of the Template for Intervention Description and Replication checklist (with most studies not applicable to the other two domains), all studies had 1–7 domains that were under-reported or not reported (see **Table 4**). Adequate reporting of the domains was as follows: intervention name and rationale (12 studies, 100.0%), materials used (6 studies, 50.0%), procedure (9 studies, 75.0%), intervention provider (8 studies, 66.7%), mode of delivery (8 studies, 66.7%), location (8 studies, 66.7%), duration and intensity (10 studies, 83.3%), expected effects (2 studies, 16.7%), and actual effects (9 studies, 75.0%).

**Table 4:** TIDieR checklist for intervention reporting.

Study ID	Brief name	Why	What (material)	What (procedures)	Who provided	How	Where	When and how much	Tailoring	Modification	How well (planned)	How well (actual)
Ewais et al. (2021) (31)	Yes	Yes	Yes	Partial	Yes	Yes	Yes	Yes	Yes	N/A	Yes	Yes
Liu et al. (2024a) (32)	Yes	Yes	Yes	Yes	No	No	No	Yes	N/A	N/A	No	No
Liu et al. (2024b) (33)	Yes	Yes	Yes	Yes	Yes	Yes	No	Yes	N/A	N/A	No	No
Memon et al. (2017) (34)	Yes	Yes	Partial	Partial	No	No	No	Partial	N/A	N/A	No	Yes
Ng et al. (2022) (35)	Yes	Yes	Partial	Yes	Yes	Yes	Yes	Partial	N/A	N/A	No	Yes
Nugent et al. (2021) (36)	Yes	Yes	Yes	Yes	Yes	Yes	Yes	Yes	N/A	N/A	No	Yes
Nyer et al. (2024) (37)	Yes	Yes	Partial	Partial	Yes	No	Yes	Yes	N/A	N/A	No	Yes
Prakhinkit et al. (2014) (38)	Yes	Yes	Partial	Yes	No	No	Yes	Yes	N/A	N/A	No	Yes
Qiu et al. (2024) (39)	Yes	Yes	Partial	Yes	Yes	Yes	Yes	Yes	N/A	N/A	No	No
Siddarth et al. (2023) (40)	Yes	Yes	Partial	Yes	Yes	Yes	Yes	Yes	N/A	N/A	Yes	Yes
Tolahunase et al. (2018) (41)	Yes	Yes	Yes	Yes	Yes	Yes	No	Yes	Yes	N/A	No	Yes
Torelly et al. (2022) (42)	Yes	Yes	Yes	Yes	No	Yes	Yes	Yes	N/A	N/A	No	Yes

Light blue for "Yes," medium blue for "Partial," dark blue for "No," and white for "N/A".

### 3.4 Research findings included in the studies

The included studies provided 21 pieces of evidence evaluating the effectiveness of MBTs on pro-inflammatory cytokine levels in patients with depression. Specifically, among the 4 pieces of evidence assessing IL-1β levels, 3 reported significant improvements following the intervention. Of the 11 pieces of evidence examining IL-6 levels, 8 reported significant improvements following the intervention. Among the 6 pieces of evidence evaluating TNF-α levels, 3 reported significant improvements following the intervention. Research findings included in the studies are presented in **Table 5**.

**Table 5:** Research findings included in the studies.

Study ID	Research findings
Ewais et al. (2021) (31)	After treatment, there was no significant difference in IL-6 levels between the experimental group and the control group
Liu et al. (2024a) (32)	After intervention, the levels of IL-1β, IL-6, and TNF-α in the experimental group were significantly lower than those in the control group
Liu et al. (2024b) (33)	IL-6 levels in the experimental group were lower than those in the control group
Memon et al. (2017) (34)	IL-6 levels were not significantly associated with treatment response on any scale
Ng et al. (2022) (35)	Compared with control, a significant reduction in IL-6 and IL-1β levels was observed in the experimental group
Nugent et al. (2021) (36)	A significant reduction was observed in IL-6 levels in the experimental group relative to the control group, while TNF-α levels did not evidence significant interactions of the experimental group by mean slope or intercept
Nyer et al. (2024) (37)	Significant differences in inflammatory biomarker levels were not found between the experimental group and the control group
Prakhinkit et al. (2014) (38)	Compared with control, a significant reduction in IL-6 levels was observed in the experimental group
Qiu et al. (2024) (39)	After treatment, the reduction of IL-6 and TNF-α levels in the experimental group was more significant than those in the control group
Siddarth et al. (2023) (40)	After treatment, there was no significant difference in TNF-α levels between the experimental group and the control group
Tolahunase et al. (2018) (41)	Compared with control, a significant reduction in IL-6 levels was observed in the experimental group
Torelly et al. (2022) (42)	Significant time effects were found for the levels of IL-1β, IL-6, and TNF-α, which, all increased following the interventions

## 4 Discussion

Given the potential risks associated with pharmacological treatments, the application of a promising complementary therapy to improve physiological health outcomes in patients with depression is particularly critical for their disease management and physical and mental recovery. The objective of this systematic review was to synthesize the latest evidence from RCTs regarding the effectiveness of MBTs on pro-inflammatory cytokines in patients with depression. The 12 RCTs provided 21 pieces of evidence involving a total of 1,058 patients with depression. The risk of bias among the included studies ranged from low to high, and their overall quality was relatively low. Among the 10 primary domains of the Template for Intervention Description and Replication checklist, all studies had 1–7 domains that were under-reported or not reported. Out of four pieces of evidence, three reported significant improvements in IL-1β levels following the intervention. Eight pieces of evidence reported significant improvements in IL-6 levels out of eleven, and three pieces of evidence reported significant improvements in TNF-α levels out of six.

Among the 21 pieces of evidence evaluated, 14 supported the positive impact of MBTs on pro-inflammatory cytokine levels in patients with depression. The mechanism underlying the antidepressant effects of MBTs can be explained from a neurobiological perspective. Specifically, MBTs such as mindfulness and meditation contribute to decreased activity of the sympathetic nervous system (SNS) and increased activity of the parasympathetic nervous system (PNS), reflecting a greater sympathetic-vagal balance. This balance is thought to reduce the body’s inflammatory response by diminishing adrenergic signaling (20, 43–45). Notably, increased activity of the SNS has been found to promote the expression of pro-inflammatory genes while inhibiting the expression of anti-viral genes (46). MBTs can reverse the impact of acute and chronic stress and reduce the activation of the SNS, which in turn helps regulate immune-related transcription (45, 47). These transcriptional regulations are primarily characterized by a reduction in NF-κB-related transcription of pro-inflammatory cytokines and an enhancement in IRF1-related transcription of innate anti-viral response (47). Accordingly, parasympathetic activation induced by the vagus nerve has been shown to increase levels of brain-derived neurotrophic factor (BDNF) (48, 49), which can inhibit glial cell activation in the central nervous system through its signaling pathways, thereby alleviating inflammatory responses (50). The increased activity of the PNS may also reduce inflammation through the cholinergic anti-inflammatory pathway (51). Additionally, alterations in cortisol production or in glucocorticoid receptor sensitivity may also modulate inflammatory processes (21). Although evidence regarding the impact of MBTs on cortisol levels remains inconsistent, several studies have found that interventions such as yoga, mindfulness, and Tai Chi can enhance glucocorticoid receptor-mediated anti-inflammatory signaling pathways, accompanied by a decrease in NF-κB activity (52–54). In terms of neural mechanisms, the activity of the ANS and the HPA axis is primarily regulated by the brain regions associated with stress or threat, including the amygdala, dorsal anterior cingulate cortex, anterior insula, and periaqueductal gray (21). Studies have shown that MBTs, such as meditation, can lead to increased thickness of the prefrontal cortex and reduce the size and activity of the amygdala (20, 43). These changes help individuals better regulate emotional responses and respond to stress or threat in a more balanced manner.

Although MBTs have shown potential in improving pro-inflammatory cytokines in patients with depression, the directionality between inflammation reduction and symptom improvement remains unclear. Clarifying this limitation is crucial for understanding the clinical implications of cytokine changes. Given that the most studies only involved short-term interventions and failed to dynamically monitor the temporal relationship between changes in inflammatory markers and clinical symptoms, we suggest conducting longitudinal studies to better infer how MBTs may alleviate depression through anti-inflammatory mechanisms. Additionally, since the included studies vary in terms of implementation parameters, including intervention period, frequency, and duration, this may lead to various dose-response relationships. Therefore, future research should establish standardized intervention protocols and conduct long-term follow-ups to examine the relationship between the efficacy of the intervention and symptom improvement. This approach will help clarify the practical value of MBTs as anti-inflammatory adjunctive strategies in clinical practice. Notably, patients’ initial condition plays a crucial role in determining the efficacy of the established intervention. Significant differences may exist among patients at baseline in terms of inflammation levels, depression severity, and treatment adherence. These differences may moderate intervention efficacy and thus influence the relationship between inflammation reduction and symptom improvement. We advocate that future research should emphasize the moderating role of patient characteristics in this context and develop personalized intervention protocols based on these features. This approach will facilitate a more comprehensive evaluation of the anti-inflammatory potential and clinical applicability of MBTs across different types and severities of depression.

In terms of the outcome, this study primarily selected IL-1β, IL-6, and TNF-α as inflammatory biomarkers associated with depression. Notably, IL-1 exists in two isoforms, IL-1α and IL-1β, both of which may possess comparable potency in activating the body’s inflammatory response (55). Although existing studies have primarily focused on the effectiveness of established interventions on IL-1β, IL-1α—being the inducible form released in an inflammatory disease state—may be more closely associated with depression than IL-1β (55, 56). Future research is recommended to further investigate the effectiveness of MBTs on additional inflammatory biomarkers in patients with depression, if sufficient evidence becomes available. This would contribute to a more comprehensive understanding of the mechanism underlying the antidepressant effects of MBTs. In terms of intervention, most of the included studies lacked adequate attention to intervention materials and adherence. On the one hand, the lack of detailed reporting on intervention materials may hinder future research from accurately replicating the original intervention process. On the other hand, heterogeneity in intervention efficacy is related to patients’ adherence levels. A lack of strategies to engage patients and maintain their participation during the intervention phase may lead to underestimation or overestimation of the intervention efficacy. Therefore, the feasibility and adherence of the intervention are important factors influencing clinical implementation. Future research should enhance transparency in the design and reporting of the intervention. We recommend that researchers follow established intervention reporting guidelines, such as the TIDieR checklist, to systematically present key information including intervention materials, implementation procedures, and parameters. This will help improve the replicability and scalability of the intervention. In addition, researchers should place greater emphasis on the assessment of adherence and strategies to promote it in their study designs. Regarding assessment, adherence indicators (e.g., actual participation frequency, completion rate) should be clearly reported, and their potential impact on intervention efficacy should be explored. Regarding strategies, it is recommended to incorporate measures designed to enhance participant motivation during the intervention implementation, such as personalized feedback and intelligent reminder systems. These approaches can help improve the feasibility and sustainability of the intervention.

The findings of this study should be interpreted in light of its limitations. First, due to the limited number of studies and available quantitative data, a meta-analysis could not be conducted. This may make it difficult to quantify the effect size of MBTs on pro-inflammatory cytokine levels in patients with depression. It is suggested that future research should examine the pooled efficacy of MBTs in this field based on a more comprehensive body of evidence. Second, this study focused exclusively on IL-1β, IL-6, and TNF-α as inflammatory biomarkers associated with depression and did not examine the effectiveness of MBTs on other inflammatory markers. Future research should expand the scope of outcome measures to more comprehensively evaluate the antidepressant effects of MBTs. Finally, the overall low quality of the included studies may affect the robustness and external validity of the findings. Future research is recommended to enhance the quality of study design, particularly in intervention materials, adherence, and outcome measures. By adhering to established intervention reporting guidelines, adopting standardized adherence monitoring, and applying uniform cytokine assays, researchers can further improve the reliability and generalizability of intervention efficacy.

## 5 Conclusion

Overall, MBTs have been widely recognized in nursing for their low risk and substantial benefits, and they hold promise as a complementary therapy to improve physiological health outcomes in patients with depression. Among the 21 pieces of evidence evaluated, 14 supported the positive impact of MBTs on pro-inflammatory cytokine levels in patients with depression. However, the included studies commonly exhibit potential limitations in terms of intervention materials, adherence, and outcome measures, which may affect the reliability and generalizability of the above findings. Therefore, future research should further examine the existing evidence to strengthen the empirical foundation for incorporating MBTs into nursing care for depression.

## Data Availability

The original contributions presented in the study are included in the article/supplementary material. Further inquiries can be directed to the corresponding author.
